# Measuring the burden of preventable diabetic hospitalisations in the Mexican Institute of Social Security (IMSS)

**DOI:** 10.1186/s12913-016-1593-1

**Published:** 2016-08-02

**Authors:** David G. Lugo-Palacios, John Cairns, Cynthia Masetto

**Affiliations:** 1 Department of Health Services Research and Policy, London School of Hygiene and Tropical Medicine, 15-17 Tavistock Place, London, WC1H 9SH UK; 2 Unidad de Planeación Estratégica Institucional, Instituto Mexicano del Seguro Social, Reforma 476, P.B., Col. Juárez, Del. Cuauhtémoc, México, D.F., CP 06600 México

**Keywords:** Preventable hospitalisations, Primary care, Diabetes, Diabetic complications, Mexico

## Abstract

**Background:**

The prevalence of diabetes among adults in Mexico has increased markedly from 6.7 % in 1994 to 14.7 % in 2015. Although the main diabetic complications can be prevented or delayed with timely and effective primary care, a high percentage of diabetic patients have developed them imposing an important preventable burden on Mexican society and on the health system. This paper estimates the financial and health burden caused by potentially preventable hospitalisations due to diabetic complications in hospitals operated by the largest social security institution in Latin America, the Mexican Institute of Social Security (IMSS), in the period 2007–2014.

**Methods:**

Hospitalisations in IMSS hospitals whose main cause was a diabetic complication were identified. The financial burden was estimated using IMSS diagnostic-related groups. To estimate the health burden, DALYs were computed under the assumption that patients would not have experienced complications if they had received timely and effective primary care.

**Results:**

A total of 322,977 hospitalisations due to five diabetic complications were identified during the period studied, of which hospitalisations due to kidney failure and diabetic foot represent 78 %. The financial burden increased by 8.4 % in real terms between 2007 and 2014. However, when measured as cost per IMSS affiliate, it decreased by 11.3 %. The health burden had an overall decrease of 13.6 % and the associated DALYs in 2014 reached 103,688.

**Conclusions:**

Resources used for the hospital treatment of diabetic complications are then not available for other health care interventions. In order to prevent these hospitalisations more resources might need to be invested in primary care; the first step could be to consider the financial burden of these hospitalisations as a potential target for switching resources from hospital care to primary care services. However, more evidence of the effectiveness of different primary care interventions is needed to know how much of the burden could be prevented by better primary care.

## Background

People with diabetes are at higher risk of developing disabling and life-threatening health problems than people without diabetes [1]. However, diabetes complications can be prevented or delayed by maintaining good control of blood glucose, blood pressure and cholesterol levels [1]. Many complications can be detected at an early stage allowing treatment that can prevent the condition becoming more serious and more costly [1]. The high economic cost and the effect on the quality of life of the population with diabetic complications impose an important preventable burden on Mexican society and on its health system [2]. The resources that are allocated to the treatment of these complications (specifically hospitalisations) are then not available for other health care interventions. This paper estimates the financial and health burden caused by potentially preventable hospitalisations due to diabetic complications in hospitals operated by the Mexican Institute of Social Security (IMSS) in the period 2007–2014.

The percentage of the Mexican population living with diabetes has increased markedly during recent decades. In 1994, the prevalence of diabetes was 6.7 %, in 2000 it grew to 7.5 % and in 2006 it reached 14.4 % [3]. The overall prevalence of diabetes among adults aged 20–79 in Mexico in 2015 was 14.7 % and Mexico ranks sixth worldwide for number of adults with diabetes (11.5 million adults) with 3.9 million having undiagnosed diabetes [1].

According to Hernández-Ávila et al. (2013), 14.3 % of the diabetic population in Mexico do not receive medical treatment; moreover, in 2012 only 25 % of those diagnosed were under metabolic control [4, 5]. The poor control of diabetes has resulted in a high number of complications: 46.9 % of diabetic patients have a diagnosis of hypertension; 47.6 % have decreased vision; 13.9 % present retinal damage; 6.6 % have lost their sight; 1.4 % receive haemodialysis because of kidney failure; 2 % have suffered an amputation and 2.8 % a cardiac arrest [5].

Diabetes is considered to be an ambulatory care sensitive condition where early diagnosis and the follow-up and monitoring of the condition can prevent exacerbation of the disease which may lead to hospitalisation [6, 7]. Moreover, timely detection and good control of diabetes is central to preventing diabetes progression and the development of vascular complications [8]. Thus, the low compliance with national diabetes control guidelines (with only 52.7 % of diabetics obtaining a blood glucose test, 14.6 % having their feet checked, and 9.6 % obtaining an HbA1c test at the time of a regular physician visit) and the high prevalence of diabetic complications suggest that the Mexican primary care system as a whole may have been overwhelmed by the diabetes epidemic [9–11].

The economic burden of diabetes in Mexico has been analysed in several studies [3, 10, 12, 13]. Barraza-Lloréns et al. (2015) estimated that in 2013 this burden, including direct and indirect costs, was MXN$362,860 million accounting for 2.25 % of GDP. Direct costs were estimated as MXN$179,495 million of which medical care for the main diabetic complications represented 87 % [13]. Lugo-Palacios and Cairns (2016) show that during 2001–2011 hospitalisation costs due to five diabetic complications (kidney failure, retinopathy, neuropathy, diabetic foot and amputation) increased by 125 %, in general hospitals run by state health ministries, reaching MXN$1,284.7 million in 2011 [2].

The health burden of diabetes in 2013 was estimated to be 1,903,650 Disability-Adjusted Life Years (DALYs) [14]. The health burden of hospitalisations due to diabetic complications in general hospitals run by state health ministries, accounted for 4.2 % of total DALYs associated with diabetes in Mexico in 2010 and increased over the period 2001–2011 by 112 % [2].

The financial cost of the hospital care provided can be considered to be a proxy for the direct economic burden of preventable hospitalisations. The health burden of these hospitalisations can be estimated by the disability suffered by patients with diabetic complications that would not have been incurred if they had received appropriate primary care [2]. Whether these hospitalisations are related to low-quality primary care, non-adherence to the recommended treatments or to easy access to secondary care through the emergency department they are, in principle, preventable at the primary level, and their presence suggests a failure of the primary care system, which includes providers, patients and health authorities, not only the primary care team.

This paper analyses the financial and health burden imposed by potentially preventable diabetic hospitalisations in IMSS hospitals. IMSS is the largest social security institution in Latin America providing health care and other social security services to more than 59 million beneficiaries from the ordinary scheme, (accounting for 49 % of the Mexican population). IMSS has recently designed and implemented a strategic plan that is intended to improve quality, health outcomes, and patient satisfaction while assuring IMSS’s financial sustainability in the short, medium, and long run [15]. Assessing the effect of these strategies on diabetic complications is, however, beyond the scope of this study. The objective of this paper is to extend the work done by Lugo-Palacios and Cairns [2] to the IMSS case by estimating the magnitude and trend of the financial and health burden associated with potentially preventable diabetic hospitalisations while avoiding double-counting of the health burden due to multiple discharges.

## Methods

This analysis follows the methodology proposed by Lugo-Palacios and Cairns [2] and uses hospital discharge data for the period 2007–2014 from six types of IMSS general hospitals: sub-zone general hospitals; zone general hospitals; regional general hospitals; and sub-zone, zone and regional hospitals with a primary care unit. Data on medical procedures, main and secondary diagnoses, as well as the code of the unit where the patient is registered to receive primary care, among other variables, were recorded for each discharge [16]. Importantly, multiple discharges for the same patient can be identified. IMSS has a well-structured information system for recording every single hospital discharge across medical units which ensures the quality of the recorded data [17].

Hospitalisations of patients 20 years or older due to five complications of diabetes (retinopathy, kidney failure, neuropathy, diabetic foot and diabetic amputations) were identified through the ICD-10 code of the main diagnosis in each case. Amputations where the main hospitalisation diagnosis was any of the diabetic codes considered in this study, were classified as diabetic amputations and included in the analysis.

To estimate the financial cost of preventable diabetic hospitalisations in IMSS hospitals this study uses the IMSS Diagnostic-Related Group (DRG) system [18]. When the ICD-10 code could be assigned to more than one DRG, the decision on which DRG to use was based on the DRG that included all the ICD-10 codes related to the complication. With the exception of diabetic amputations, the DRG costs were taken from the IMSS Medical-Economic Forms where cost estimates for each DRG are reported. Since diabetic amputations are defined as those hospitalisations where the main diagnosis was any diabetic ICD-10 code where the patient suffered an amputation, only the cost of the surgical procedure was considered as this intervention represents additional costs not previously accounted for in the DRG cost. The latter cost was obtained from the inter-institutional maximum referral tariffs [19]. Table 1 shows the DRG classification and costs for all the ICD-10 codes considered in this analysis. It is worth noting that the codes considered for all the complications analysed (including kidney failure) are only those related to diabetes. IMSS-DRG cost data are only available for 2013, thus, it was necessary to assume that IMSS-DRG costs only changed due to inflation during the study period.

**Table 1:** Diabetic ICD-10 codes and DRG classification

ICD-10 of diabetic complications		DRG	IMSS DRG 2013 Cost (MXN)
Kidney failure	E10.2, E11.2, E12.2, E13.2, E14.2	698 – Other kidney and urinary tract diagnostics with major complications	71 066
Retinopathy	E10.3, E11.3, E12.3, E13.3, E14.3	125 – Other eye disorders	22 820
Neuropathy	E10.4, E11.4, E12.4, E13.4, E14.4	074 – Cranial and peripheral nerve disorders with no major complications	37 494
Diabetic foot	E10.5, E11.5, E12.5, E13.5, E14.5	301 – Peripheral vascular disorders	46 057
Amputation	Any Diabetic code + CIE-9CM: 84.1, 84.10, 84.11, 84.14, 84.15, 84.17, 84.19	Low limb amputation secondary to diabetic foot	58 831
All diabetic hospitalisations ICD-10 codes			
E10.9, E11.9, E12.9, E13.9, E14.9, E10.0, E10.1, E10.6, E10.7, E10.8, E11.0, E11.1, E11.6, E11.7, E11.8, E12.0, E12.1, E12.6, E12.7, E12.8, E13.0, E13.1, E13.6, E13.7, E13.8, E14.0, E14.1, E14.6, E14.7, E14.8, E10.5, E11.5, E12.5, E13.5, E14.5, E10.3, E11.3, E12.3, E13.3, E14.3, E10.2, E11.2, E12.2, E13.2, E14.2, E10.4, E11.4, E12.4, E13.4, E14.4			

*Source*: Lugo-Palacios and Cairns [2]

The estimation of the health burden assumes that patients would not have experienced complications if they had received appropriate primary care and computes the associated DALYs. Disability weights for diabetic foot, neuropathy, kidney failure – stage IV, amputation of toe, amputation of one leg, and amputation of both legs were taken from the Global Burden of Disease Study 2010 [20]. The weight for retinopathy-blindness was taken from the Global Burden of Disease 2004 Update, since the 2010 version did not report a weight for this condition [21]. Due to lack of detail concerning the severity of the condition of hospitalised patients (e.g. degree of kidney failure or seriousness of retinopathy) and the absence of disability weights for different severity levels, only one level of disability (equal to the available weight in each case) is considered for patients whose main hospitalisation diagnosis was kidney failure, retinopathy, neuropathy and diabetic foot. WHO data on the life expectancy at age with the lowest mortality observed worldwide are used to compute the Years of Life Lost (YLL) and Years Lived with Disability (YLD) [22, 23].

Some hospitalised patients whose main diagnosis was kidney failure, neuropathy, retinopathy and diabetic foot also suffered amputations. Hence, to avoid double counting of deaths while computing YLL it was necessary to define the variable “net amputation” indicating those diabetic amputations in which the main cause of hospitalisation was none of the other complications; therefore, amputation YLL are based on net amputations. However, when computing YLD the total number of people suffering amputations was used, since amputations will contribute to their disability; in this case, patients with diabetic foot without amputations (net diabetic foot) were used to compute diabetic foot YLD.

## Results

Table 2 shows the composition of hospitalisations due to diabetic complications in IMSS general hospitals during 2007–2014. A total of 322,977 hospitalisations met the described criteria, of which hospitalisations due to kidney failure and diabetic foot represent 78 %. Hospitalisations due to diabetic complications increased by 10.3 % over this period. Total hospitalisations caused by diabetic complications per 10,000 IMSS affiliates (not shown), decreased by 9.8 % from 7.91 in 2007 to 7.13 in 2014, reaching a maximum (8.15) in 2008 and a minimum (6.96) in 2013.

**Table 2:** Diabetic preventable hospitalisations in IMSS 2007–2014. Hospital discharges

Hospital discharges									
	2007	2008	2009	2010	2011	2012	2013	2014	Total
Kidney failure	15 369	16 744	14 635	14 893	15 400	15 925	14 977	14 353	122 296
Retinopathy	2 393	1 554	1 690	1 597	1 764	1 446	1 824	2 178	14 446
Neuropathy	720	690	683	650	691	602	650	660	5 346
Diabetic foot	14 000	14 608	14 697	16 433	16 816	16 818	17 070	17 759	128 201
Amputation	6 001	6 285	6 226	6 646	6 571	6 560	6 889	7 510	52 688
TOTAL	38 483	39 881	37 931	40 219	41 242	41 351	41 410	42 460	322 977
Multiple discharges for the same complication in the same year									
	2007	2008	2009	2010	2011	2012	2013	2014	Total
Kidney failure	2 854	3 369	2 521	2 678	2 865	2 959	2 772	2 685	22 703
Retinopathy	376	249	148	124	256	274	373	461	2 261
Neuropathy	22	23	15	22	13	15	16	17	143
Diabetic foot	1 939	2 013	2 035	2 294	2 425	2 420	2 557	2 573	18 256
Amputation	422	453	433	457	455	462	472	546	3 700
TOTAL	5 613	6 107	5 152	5 575	6 014	6 130	6 190	6 282	47 063
At least one admission for the same condition in previous years (plus multiple discharges in the same year)									
	2007	2008	2009	2010	2011	2012	2013	2014	Total
Kidney failure	-	4 094	3 493	3 709	3 924	4 151	3 937	3 822	27 130
Retinopathy	-	323	237	255	350	363	474	575	2 577
Neuropathy	-	30	21	34	21	22	26	30	184
Diabetic foot	-	2 752	3 060	3 686	4 029	4 166	4 394	4 622	26 709
Amputation	-	632	724	851	876	914	987	1 104	6 088
TOTAL	-	7 831	7 535	8 535	9 200	9 616	9 818	10 153	62 688
Number of IMSS affiliates per year									
2007	2008	2009	2010	2011	2012	2013	2014		
48 650 488	48 909 706	49 134 310	52 310 086	54 906 396	57 475 897	59 511 963	59 487 144		

*Source*: Authors using data from IMSS (2015) and INEGI (2015) [16, 25]

From Table 2 it can also be observed that the percentage of multiple admissions/discharges for the same complication in the current year oscillates around 15 % of the total. Only 3 % of diabetic neuropathy hospitalisations per year fall into this classification. Whereas, multiple discharges in the same year are more important in the case of kidney failure and retinopathy, accounting for more than 20 % of hospitalisations in some years.

Hospitalisations of patients that have been admitted at least once for the same condition in previous years increased their share throughout the period, but this increase is not the same for all conditions. The most important increment in the share of these multiple discharges is observed in amputations which grew 46 % from 10.1 % of amputations in 2008 to 14.7 % in 2014; neuropathy was the complication whose multiple discharges share increased the least (4.5 %). An increase in multiple discharges reflects the fact that the probability of having at least one hospitalisation in previous years increases over time and, of course, an unknown proportion of patients in the first year of analysis (2007) were hospitalised in previous years; nevertheless, these data give an indication of the extent to which diabetes is being controlled over time.

### Financial burden

Tables 3 and 4 show the estimated financial costs of hospitalisations resulting from diabetic complications. These costs increased by 8.4 % in real terms between 2007 and 2014. However, when measured as cost per IMSS affiliate, the estimated costs decreased by 11.3 % from MXN$41.5 in 2007 to MXN$36.8 in 2014. The hospitalisation costs of kidney failure, retinopathy and neuropathy decreased by 7, 9 and 8 %, respectively, while those for diabetic foot and amputations increased by more than 25 %. Despite these changes, kidney failure remains the most important cause of preventable hospitalisation costs, accounting for 43 % of costs in 2014.

**Table 3:** Financial ACSH cost (2011 Million MXN)

	2007	2008	2009	2010	2011	2012	2013	2014
Kidney failure	1 014.32	1 105.08	965.86	982.87	1 016.34	1 051.01	988.41	947.21
Retinopathy	50.71	32.93	35.81	33.84	37.38	30.64	38.65	46.16
Neuropathy	25.07	24.03	23.78	22.63	24.06	20.96	22.63	22.98
Diabetic foot	598.81	624.83	628.61	702.85	719.24	719.34	730.09	759.55
Amputation	327.87	343.39	340.15	363.09	359.00	358.41	376.37	410.29
TOTAL	2 016.78	2 130.26	1 994.22	2 105.29	2 156.03	2 180.36	2 156.15	2 186.18

**Table 4:** Financial costs per IMSS affiliated (2011 MXN)

	2007	2008	2009	2010	2011	2012	2013	2014
Kidney failure	20.8	22.6	19.7	18.8	18.5	18.3	16.6	15.9
Retinopathy	1.0	0.7	0.7	0.6	0.7	0.5	0.6	0.8
Neuropathy	0.5	0.5	0.5	0.4	0.4	0.4	0.4	0.4
Diabetic foot	12.3	12.8	12.8	13.4	13.1	12.5	12.3	12.8
Amputation	6.7	7.0	6.9	6.9	6.5	6.2	6.3	6.9
Total	41.5	43.6	40.6	40.2	39.3	37.9	36.2	36.8

*Source*: Authors using data from IMSS (2015) [16]

### Health burden

The estimated DALYs associated with diabetic complications are presented in Table 5. Overall, as opposed to the financial costs, DALYs decreased by 13.6 % from 2007 to 2014; however, in the last 3 years of the period, they increased slightly by 3.4 %. The latter is explained mainly by the 44 % increase observed in the DALYs associated with diabetic retinopathy after 2012.

**Table 5:** Disability Adjusted Life Years (DALYs) associated with diabetic preventable hospitalisations. IMSS 2007–2014

Complications	2007	2008	2009	2010	2011	2012	2013	2014
Kidney failure	67 139	67 341	60 409	57 960	60 578	61 261	58 265	54 683
Retinopathy	33 931	20 350	23 777	22 021	23 701	18 343	22 509	26 481
Neuropathy	2 300	2 317	2 278	2 167	2 230	1 950	2 211	2 117
Diabetic foot	4 173	5 017	5 459	5 869	4 839	4 952	4 005	4 611
Amputation	12 508	12 917	13 052	13 561	13 209	13 813	14 634	15 796
TOTAL	120 051	107 941	104 975	101 578	104 557	100 320	101 625	103 688

*Source*: Authors using data from IMSS (2015)

During the whole study period, the DALYs associated with kidney failure have always represented more than 50 % of the estimated total, reaching a peak of 62 % in 2008.

When disaggregating DALYs into YLD and YLL (not shown, but available upon request), the gap that grew in 2007–2008 between the YLD associated with kidney failure and those associated with retinopathy, following the important drop in retinopathy admissions, narrowed in recent years due to a sustained fall in kidney failure YLDs and a steep increase in retinopathy YLDs during 2012–2014. Amputation YLDs as a share of total YLDs rose in successive years from 13 % in 2007 to 20 % in 2014.

**Fig. 1:**
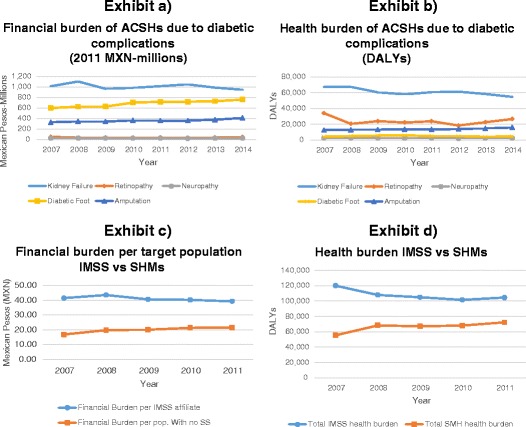
Financial and health burden of diabetic preventable hospitalisations 2007–2014

Figure 1 (exhibits a and b) presents the financial and health burden over time for each of the complications. It shows that the financial and health burdens for kidney failure have decreased throughout the period. While the gap between the kidney failure health burden and that of the other diabetic complications is still considerable, the difference in the financial burden has importantly narrowed due to the increase in both the absolute and relative importance of the diabetic foot and amputation hospitalisation costs.

## Discussion

This study identifies potentially preventable hospitalisations due to five diabetic complications (kidney failure, retinopathy, neuropathy, diabetic foot and amputation) from 2007 to 2014 and estimates the associated financial and health burden. These hospitalisations increased by 10.3 % during the study period and the estimated financial costs of hospitalisations resulting from diabetic complications increased by 8.4 % in real terms, reaching MXN$2,186 million in 2014; when measured as costs per IMSS affiliate the estimated costs decreased by 11.3 % from MXN$41.5 in 2007 to MXN$36.8 in 2014. The total health burden, expressed in DALYs associated with these conditions, decreased by 13.6 %.

Table 6 compares the findings of this paper with the burden estimated for the general hospitals run by the state health ministries (SHMs) [2]; the financial and the health burden of preventable diabetic hospitalisations in IMSS per capita in 2011 is higher by 82 and 45 %, respectively. The difference in health burden may be greater since the present study avoids double counting DALYs when patients are admitted more than once for the same cause and/or if patients died during their hospitalisations in a given year, unlike the data from the SHMs.

**Table 6:** Comparison IMSS vs State Health Ministries (SHMs)

	IMSS	SHMs
2011 financial burden per capita^a^	39.3	21.6
2011 health burden (DALYs per 10 000 population)	19.04	12.11
2001–2011 financial burden change (%)	-	95.4
2001–2011 health burden change (%)	-	111.8
2007–2014 financial burden change (%)	−11.3	-
2007–2014 health burden change (%)	−13.6	-
2007–2011 financial burden change (%)	−5.3	28.2
2007-2011 health burden change (%)	−12.9	30.5

*Source*: Authors using data from IMSS (2015)

^a^The financial burden is expressed in per capita terms. In the case of IMSS, it is per IMSS beneficiary and in the case of SHMs is per person with no social security (major demanders of their services). The financial burden changes reported in this table were computed using per capita values while the health burden changes were computed using absolute values

Differences between IMSS and the SHMs in the trend of the burden of diabetic complications arise for two main reasons. First, increases in the estimated prevalence, diagnosis, and treatment of diabetes are largely attributable to the expansion of health insurance pushing preventable hospitalisations up among previously uninsured whose poorly controlled/unknown diabetes hindered the ability of primary care to avoid hospitalisations [7, 24]. While SHMs had to face the increase in the demand for health services following this health insurance expansion, IMSS coverage has remained relatively stable during the last decade [25]. Second, as Arredondo and De Icaza (2011) show, and consistent with the findings presented here, IMSS costs per diabetic patient treated are 80 % higher mainly due to differences in case management protocols, in productivity standards, in quality standards and in the cost of inputs [26].

Apart from differences in the magnitude of the burdens, another important difference is the trend that each burden follows. In the case of SHMs, the rate of diabetic complications, the financial burden, the financial burden per person with no social security, and the health burden increased over the study period, while for IMSS only the financial burden from diabetic foot and amputation hospitalisations increased. The financial burden of diabetic foot and amputation increases among patient groups in IMSS and SHMs. Exhibits c and d in Fig. 1 show the comparison of the financial burden per target population and the total health burden of IMSS and of SHMs for 2007–2011. Both IMSS burdens show a decreasing trend while the opposite is observed for the SHMs.

All major complications of diabetes can be prevented or delayed by good control of blood glucose, blood pressure and cholesterol levels [1]. This requires the patient to be well-informed regarding management of their condition, as well as access to insulin, oral medications and monitoring equipment. People with diabetes should be supported by a well-educated health work force and health systems that provide regular blood tests and eye and foot examinations [1]. In addition, it should be noted that, paradoxically, as people with diabetes live longer they become more likely to suffer diabetic complications; therefore, it is important to develop new strategies that can prevent the onset or progression of diabetic complications [27]. During recent years, IMSS has implemented a number of strategies to improve the control of chronic conditions among its beneficiaries, especially those with diabetes. Evaluation of these strategies is beyond the scope of this paper; however, the evidence presented indicates that the rate of hospitalisation due to complications of diabetes per IMSS affiliate, the financial burden per IMSS affiliate, and the associated health burden has decreased during the period studied. Since the absolute financial burden increased more than 8 %, there are likely to be opportunities to shift resources from expensive hospital care to more cost-effective primary care interventions; especially since the hospitalisation costs for diabetic foot and amputations are increasing, and these are avoidable with good diabetes management, specifically, with regular foot examination [1].

Improving the quality of and effective access to public primary care services is crucial to ensure appropriate diabetes management as it is worrying that one in eight users state that they would avoid these services in the future mainly because of unacceptable waiting times, mistreatment or no improvement in their condition [28]. Furthermore, it is necessary to tackle misconceptions that primary care is basic health care, health care for the poor or rural health care, among the sector of the population that still prefers hospital over primary care [29].

The fact that the total health burden decreases while both the number of preventable hospitalisations and the total financial burden increase is due to the way in which DALYs are computed. The contribution of a patient to the DALYs count of a specific condition only takes into account the first hospitalisation of the patient for the same cause over the period in order to avoid double-counting. Therefore, multiple admissions of the same patient for the same cause do not contribute to the health burden. However, every hospitalisation, whether or not it is the first or a subsequent one, represents a cost to the hospital. Furthermore, the importance of avoiding double-counting of the health burden associated with multiple discharges for the same condition over time is clear when 24 % of total hospital discharges due to diabetic complications in 2014 do not contribute to the DALYs count.

This analysis is subject to a limitation present in early work [2]. Since the severity of the condition for which patients were hospitalised is not recorded, and there is a lack of disability weights for different severity levels of kidney failure and retinopathy, all kidney failure and retinopathy admissions were assumed to have the same severity level: stage IV and blindness, respectively. This assumption causes an overestimation of the associated DALYs and should be taken as the upper bound of the health burden associated with preventable diabetic hospitalisations. As opposed to Lugo-Palacios and Cairns [2], by tracking multiple discharges from the same patient over the study period, the present paper avoids the double-counting of DALYs when patients are admitted more than once for the same cause throughout the period and/or if patients died in any of their hospitalisations in a given year. A second limitation is that both dimensions of the burden associated with preventable diabetic hospitalisations only consider affiliates receiving care in IMSS hospitals; however, affiliates seeking care elsewhere or not seeking care at all are not taken into account (around 30 % of IMSS affiliates seek primary care from the private sector [29]). In addition, IMSS-DRG costs do not necessarily represent what IMSS hospitals are actually spending on each treatment, but rather are used as a benchmark, and IMSS-DRG costs do not consider rural-urban nor big-small city price differentials [2]. Nevertheless, these costs are the most robust hospital cost data available for IMSS.

## Conclusions

Timely and effective primary care services that prevent the development or the exacerbation of the condition can reduce the burden of preventable hospitalisations. The resources used to treat avoidable hospitalisations could, in principle, be used to fund more and better primary care services. However, more evidence is required concerning which strategies are best for preventing hospitalisations and how much of the burden could be prevented by better primary care. This study might be seen as setting an upper limit to the potential benefit from improving primary care. IMSS is currently integrating primary care and hospital data at the patient level. Consequently, it should be possible to obtain a better understanding of the scope for primary care to prevent hospitalisations. The improvement of record linkage among levels of care and among all Mexican health care institutions through the patient clinical-electronic file is crucial in the design of a new integrated primary care system that could provide opportunities to reduce the financial and health burden of diabetic complications.

## Abbreviations

DALYs, Disability Adjusted Life Years; DRG, Diagnostic-Related Group; HbA1c, Glycated haemoglobin; ICD-10, International Statistical Classification of Diseases and Related Health Problems 10th Revision; IMSS, Instituto Mexicano del Seguro Social (Mexican Institute of Social Security); MXN, Mexican Pesos; SHMs, state health ministries; YLD, Years Lived with Disability; YLL, Years of Life Los
